# The Mechanism of Household Waste Sorting Behaviour—A Study of Jiaxing, China

**DOI:** 10.3390/ijerph19042447

**Published:** 2022-02-20

**Authors:** Qiao Liu, Qianhui Xu, Xin Shen, Bowei Chen, Sonia Sadeghian Esfahani

**Affiliations:** 1 College of Economics and Management, Shanghai Ocean University, Shanghai 201306, China; q_liu@shou.edu.cn (Q.L.); chen15901819068@gmail.com (B.C.); 2 ICT School, College of Science and Engineering, University of Tasmania, Hobart 7005, Australia; sonia.sadeghianesfahani@utas.edu.au

**Keywords:** waste sorting, theory of planned behaviour, value-belief-norm theory, structural equation modelling, China

## Abstract

Economic growth and rapid urbanization have resulted in various urban issues related to sustainable development in emerging economies such as China. Nowadays, two-thirds of China’s cities are besieged by waste and one-fourth of the cities have no space to build landfills. China is embarking on a top-down waste sorting revolution, in which residents’ awareness and behaviour of participation are fundamental to the success of garbage classification. The purpose of this paper is to understand residents’ waste sorting behaviour and identify the influencing factors in China. The subjects of this study are urban and rural residents in Jiaxing, where local government has begun to encourage waste classification but has not yet legalized it. With the integration of the theory of planned behaviour (TPB) and value-belief-norm theory (NAM), this study represents a “motivation-intention-behaviour” theoretical model for the systemic illustration of the antecedents of household waste sorting behaviour. A total of 541 questionnaires were administered in person in households in Jiaxing, China. Structural equation modelling with partial least squares was applied to analyse empirically. The results show that attitudes (ATT), subjective norm (SN), and perceived behavioural control (PBC) have a significant positive effect on the intention of household waste sorting (WSI), and the perceived policy effectiveness (PPE) has a positive and significant effect on the attitudes and waste sorting intention. The waste sorting intention has a positive and significant effect on waste sorting behaviour (WSB). In addition, individual characteristics have a significant impact on waste sorting behaviour, where respondents of women, higher income, and middle and old age are more willing to implement waste sorting behaviours. This study theoretically contributes to the literature by improving the understanding of the determinants of household solid waste sorting behaviour. It also provides several recommendations to improve existing policies at the practitioner level. These recommendations can be valuable references for waste management in China and other emerging economies.

## 1. Introduction

The world generates at least 1.47 billion tons of municipal solid waste each year [1]. China has become the world’s largest waste producer since 2004 as reported by the World Bank [2]. With the acceleration of the urbanization process and the gradual improvement of people’s living standards, the amount of domestic waste generated in Chinese cities has been increasing year by year. A report in 2019 revealed that China produces 10 billion tons of garbage every year, with an annual stock of 6 billion tons and 500 million square meters of farmland occupied [3]. The rate of garbage explosion has outstripped the garbage disposal capacity of urban areas in China. Statistics from the Ministry of Housing and Urban-Rural Development of China show that over two-thirds of China’s cities are besieged by waste and one-fourth of the cities have no space to build landfills. Waste disposal is a systematic engineering, and effective waste classification management can maximize the utilization of domestic waste resources, reduce the amount of waste disposal, and improve the living environment of residents [4]. Nowadays, waste sorting has been upgraded to a national strategy since the National Development and Reform Commission of China issued the “Plan for the Mandatory Waste Classification System” in June 2016. On 1 July 2019, Shanghai took the lead in entering the era of obligatory waste sorting. At the end of 2020, there were 46 key cities in the country being piloted; the goal is to build a waste sorting and processing system.

Waste sorting emphasizes the whole process. Good sorting of front-end residents is the key to mid-end collection and transportation, back-end disposal, and resource utilization [1]. It has also become an important way out for the implementation of the entire waste sorting strategy and the plight of waste siege. Residents are the producers of domestic waste in the front-end and the subjects of classified distribution [1,4]. In this top-down waste sorting revolution of China, residents’ awareness and behaviour of participation are fundamental to the success of garbage classification. Therefore, it is worthy of in-depth study to explore the influencing factors of household solid waste sorting behaviour.

Household waste sorting belongs to one of the fields of public environmental behaviour selection. Prior studies include two perspectives of economics and psychology. From the perspective of economics, the public irrational thinking is the starting point to explore the motivation mechanism of participation, such as the subject’s interest game and differentiated responsibilities [5], etc., indicating the externality nature of garbage classification [6]. Second, from the psychology point of view, garbage classification is mostly based on the factors of behaviour influencing, such as residents’ perception and individual characteristics [1,4,7].

The purpose of this work is to understand residents’ waste sorting behaviour and identify the influencing factors in China. The subjects of this study are urban and rural residents in Jiaxing, where local government has begun to encourage waste classification but has not yet legalized it. This study proposes a conceptual model of the behaviour mechanism of household waste sorting based on an integrated framework of the theory of planned behaviour (TPB) and the value-belief-norm (VBN) theory. Questionnaires were used to explore the factors influencing waste sorting behaviour.

This paper begins with the specification of the contextual model and with the analysis of the relevant literature to develop the research hypotheses. Then, the research methodology is highlighted and the materials and methods are described. Next, the results of the study are presented and the determinants of household solid waste sorting behaviour are discussed. Finally, the conclusions of this research are presented, highlighting the main implications and limitations of the research. It also provides several recommendations to improve existing policies at the practitioner level. These recommendations can be valuable references for waste management in China and other emerging economies.

## 2. Literature Review and Hypotheses

The theory of planned behaviour is an important theoretical model for studying public behaviour choices from a micro-perspective. A TPB model includes three main factors, namely attitudes (ATT), subjective norms (SN), and perceived behaviour control (PBC) to predict individual behaviour intentions [8,9].

As one of the mature theories of public behaviour research, a TPB has also obtained empirical support in the research field of waste sorting behaviour domestic and foreign.

Ajzen defined ATT as the positive or negative cognitive representation of an individual’s performance for a particular behaviour [8,9]. Many studies [4,7,10,11,12,13,14,15] have reported the significantly positive influence of attitudes on intentions of pro-environmental behaviours. Zhang et al. [4] claim that the attitudes of residents in Shenzhen and Tianjin, China, are an important factor in promoting their intention to participate in sorting and recycling. This is also supported in the research of Jia et al. [10] with the micro-survey data of rural residents in Shaanxi province, China. Alhassan et al. [7] also point out ATT as one of the determinants of the garbage separation behaviour intention in Ghana. According to these, the following hypothesis is proposed.

**H1:**  *Residents’ attitude (ATT) has a positive and significant influence on the waste sorting intention (WSI).*

SN is defined as the belief about others’ attitudes toward behaviour and the perception of the social pressure for an individual’s intention to take a particular action [11]. In the perspective of waste classification, SN refers to a person’s belief about whether significant others think he or she should engage in the source sorting behaviour [12,13]. Some studies [4,12,13] indicate that sorting and recycling waste by neighbours or companions can drive other residents’ behaviour. Alhassan et al. [7] also consider SN as an important factor to determine the households’ source separation behaviour intention in Ghana. In China, the atmosphere of garbage sorting is forming in the whole society [1,2,4]. In Shanghai, for example, picture books on waste classification have been introduced to kindergartens, and community volunteers have been helping each other with the trash cans [2]. Neighbours have been helping each other. The following hypothesis is thus proposed.

**H2:**  *Subjective norms (SN) have a positive and significant impact on waste sorting intention (WSI).*

PBC is defined as the perceived ability to control an individual’s particular behaviour [8,13]. According to Ofstad et al. [12], this belief about one’s ability to perform a behaviour will trigger his or her desire to act in the classification and recycling of waste. Residents who have more time, energy, funds, and other self-controllable factors are more likely to have a willingness to sort waste and take action [1,14]. In reality, some facilities such as smart waste classification and collection systems [2] can enhance personal PBC. The following hypothesis is thus proposed.

**H3:**  *Perceived behaviour control (PBC) has a positive and significant impact on waste sorting intention (WSI).*

Behavioural intention can encourage others to adopt particular behaviour in the future [8,9]. Some scholars [4,12,13] claim there is a relatively stable causal relationship between waste sorting intention and implementation behaviour. The following hypothesis is thus proposed.

**H4:**  *Household waste sorting intention (WSI) has a positive impact on waste sorting behaviour. (WSB).*

The value-belief-norm theory (VBN) is an important theory in the study of public environmental behaviours, in which key variables perceived policy effectiveness, and have been proven to have good explanatory power for public environmental behaviour intentions such as recycling [1,14,15,16]. Residents have positive recognition of the results of the implementation of the waste sorting policy and recognise that the measures taken by the government are implemented based on the optimisation and protection of the residents and the urban environment [16]. They are some effective policies that can be implemented. The residents’ perception of the policy implementation has a direct impact on individual attitudes [15,16]. Shen et al. [1] claim that the government improves residents’ beliefs to adopting waste sorting behaviour by implementing effective policies. This is also supported in the study of Liao et al. [16] in terms of the perceived effectiveness of both the inducement policy and attitude. In this study, PPE refers to residents’ belief in the government policy effectiveness of waste classification. PPE has a direct influence on WSI as an effective policy can act as an incentive for people to sort or recycle: If people perceive a policy to be effective, their intentions to classify waste will be increased [1,14,15,16]. Therefore, the following hypotheses are put forward:

**H5:**  *Perceived policy effectiveness (PPE) has a positive and significant impact on residents’ attitudes (ATT).*

**H6:**  *Perceived policy effectiveness (PPE) has a positive and significant impact on waste sorting intention (WSI).*

The model of the theory of planned behaviour has no specific requirements on the selection of external variables [17,18]. Researchers often add individual characteristics, task characteristics, and other exterior factors to investigate according to their own research needs [10,19,20,21,22]. In this study, we select external variables such as gender, age, education background, income, and living area and proposes a hypothesis as follows:

**H7:**  *Individual characteristics of residents have a positive and significant impact on the decision-making about waste sorting.*

**Figure 1:**
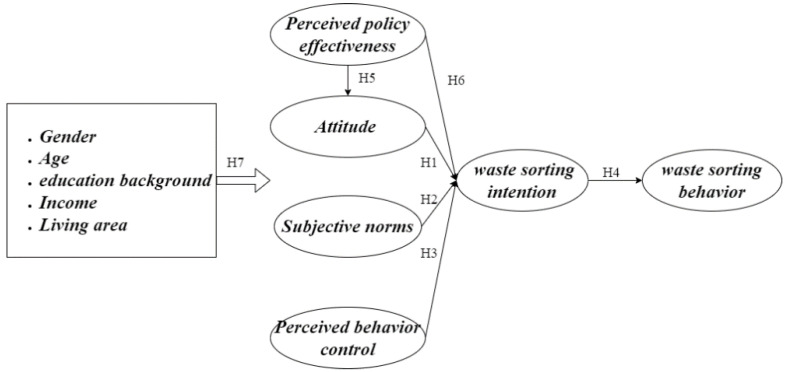
Theoretical model for decision making on household waste sorting.

Based on the above assumptions, the research concept model is shown in Figure 1.

## 3. Materials and Methods

### 3.1. Measurements

The questionnaire is developed based on the existing relevant literature. Table 1 details the questionnaire. The questionnaire is divided into two parts. The first part includes the basic information of the respondents, including gender, age, income, education background, living area while the second section of the questionnaire focuses on the behaviour of household waste sorting. In the second part of the items, the TPB scale primarily refers to the scale designed by Ajzen et al. [8,9], and the dimension of perceived policy effectiveness mainly refers to the scale of Wan et al. [15]. The questionnaire uses a 7-point Likert scale to measure the degree of identity of the interviewees, from 1 to 7, representing the degree of identity from low (strongly disagree) to high (strongly agree).

### 3.2. Data Collection

The subjects of this study are urban and rural residents in Jiaxing, China. Located in the Yangtze River Delta city cluster, Jiaxing City, Zhejiang Province, is an important city in the Shanghai Metropolitan Circle, a central city in the Greater Bay Area of Zhejiang, and a subcentral city in the Hangzhou Metropolitan Circle. Its permanent population in 2019 was 4.65 million. According to the sample size estimation table, the number of samples required for this survey is 384. Jiaxing has two municipal districts (Nanhu District, Xiuzhou District), three county-level cities (Haining City, Pinghu City, Tongxiang City), and two counties (Jiashan County, Haiyan County). This study adopts a population ratio quota sampling method and questionnaires will be distributed from June 1 to June 30, 2021, and a total of 600 questionnaires collected. After deducting 59 invalid questionnaires with the same answer options, 541 questionnaires were effectively returned. The effective questionnaire recovery rate was 90.17%.

### 3.3. Data Analysis

In this study, IBM, SPSS and Structural Equation Modelling (SEM) were used for analysis, and the software versions used were SPSS17.0 and SmartPLS3.2.9. The analysis content consists of three parts: (1) Measurement model analysis: to analyse the reliability and validity of each variable dimension; (2) structural model analysis: to understand the significance of independent variables (attitudes, subjective norms, perceived behaviour control, perceived policy effectiveness) through intermediary variables (behaviour intentions) to dependent variables (waste sorting behaviour) and verify the validity of relevant assumptions. (3) In addition, some individual characteristics such as gender, age, income, level of education, and living area are used as moderating variables to further develop a multigroup structural equation model analysis. These variables are analysed to verify the impact of individual characteristics of the household waste sorting behaviour of domestic household waste.

## 4. Results

### 4.1. The Measurement Model Analysis

In this study, SEM applied is a technique of multivariate statistical analysis. Partial least squares (PLS) is a common SEM method to confirm the effectiveness of tool structures (measurement model or outer model) and to evaluate the structural relationship (structure model or inner model) [23,24,25]. PLS works well with nonnormal distributions and smaller sample sizes [26,27].

Measurement model analysis includes reliability and validity analysis. Fornell and Larcker [23] proposed the evaluating measurement scales as follows: all factor loadings should be significant and exceed 0.5; construct reliabilities should exceed 0.7, and (c) the average variance extracted (AVE) by each construct should exceed the amount of measurement error variance (AVE > 0.5).

First, a reliability test is performed. Factor loadings of the observed variables used in each latent variable are shown in bold in Table 2, and all satisfy research requirements greater than 0.5. On this basis, the internal consistency test is carried out. The Cronbach’s alpha coefficients of ATT, SN, PBC, WSI, PPE, and WSB are all greater than 0.7 (0.895, 0.865, 0.773, 0.901, 0.929, 0.878, respectively). The composite reliability (CR) is between 0.903–0.943 (0.923, 0.903, 0.850, 0.927, 0.943, 0.916, respectively), which are all higher than the basic requirement of 0.7, indicating that the model has acceptable reliability. Secondly, convergence validity and discriminative validity tests are performed. The average variance extraction (AVE) value of all constructs is between 0.650 and 0.732 (0.705, 0.650, 0.662, 0.716, 0.704, 0.732, respectively). As shown in Table 2, the factor loadings of each dimension are larger than the correlation coefficients between the variable and other latent variables, which is in line with the index proposed by Fornell-Larcker [23] to determine the discriminative validity using cross-loading, indicating that the research model has acceptable validity.

### 4.2. The Structural Model Analysis

The second part is for the structural model that displays the relationships between these constructs [24]. The structural model aims to analyse the conceptual model’s ability to predict the variance of the dependent variables and independent variables. The study uses the bootstrapping method to calculate the significance of the path. This method is to use the limited 541 sample data to re-establish a new 5000 weighted sample that is representative of the distribution of the maternal sample through repeated sampling [25,28]. After testing, each research hypothesis satisfies the requirements at a significance level of 5%, the hypothesis has been verified, and the theoretical model of the behaviour mechanism of household waste sorting is established. Attitudes, subjective norms, perceived behaviour control, and perceived policy effectiveness among the variables in the research model all have significant positive effects on the household waste sorting intention, and the household intention for waste sorting significantly improves the sorting behaviour.

Hypotheses 1, 2 and 3 examine the effects of attitudes, social norms, and perceived behaviour control on waste sorting intention, respectively. It was shown that attitudes (β = 0.198, *p* < 0.001), social norms (β = 0.314, *p* < 0.001) and perceived behaviour control (β = 0.162, *p* < 0.001) were significantly related to waste sorting intention. Hypotheses 4 examines the effects of waste sorting intention control on waste sorting behaviour. waste sorting intention was significantly related to waste sorting behaviour (β = 0.629, *p* < 0.001). Hypotheses 5 and 6 examine the effects of perceived policy effectiveness on attitudes and waste sorting intention. Perceived policy effectiveness was significantly related to attitudes (β = 0.831, *p* < 0.001) and waste sorting intention (β = 0.308, *p* < 0.001). The results of these analyses are present in Table 3.

### 4.3. Multi-Group Analysis

To verify the influence of individual characteristics on the behavioural mechanism of household waste sorting, external indicators such as gender, age, income level, education level, and living area are used as the moderating variables, and the multi-group structural equation model analysis is further carried out. These PLS-MGA results are shown in Table 4 (for the subgroups of gender, income and age) and Table 5 (for the subgroups of education back ground and living area). Monthly income is divided into low income and high income according to the standard of 5000 Chinese yuan. All respondents were over 18 years old, and two groups were divided according to the age of 40 years old as young and old subgroups. Taking higher education (college and undergraduate and above) is considered the dividing standard and it is divided into two groups: non-higher education and higher education. Depending on the living area, there are two groups: urban and rural subgroups. The analysed results for each group are consistent with the overall verification results, but the impacts caused by different characteristics within each group are different. Therefore, the individual characteristics of residents have a positive and significant impact on the decision-making about waste sorting, and H7 has been verified.

## 5. Discussion

Through the above analysis of the results, we found that the more positive attitude of the individual residents, the stronger the control of self-behaviour and the clearer the intention of waste sorting behaviour, which is consistent with the research conclusions of Zhang et al. [4], Ofstad et al. [12], and Xu et al. [17]. Subjective norms have the greatest influence on the household waste sorting intention, which is consistent with the research viewpoints of Shen [1] but is contrary to the study results of Wen et al. [14] and Xu et al. [17]. The latter two believe that subjective norms have a weaker impact on the waste sorting behaviour of urban residents. The possible reason is that their research was conducted before July 2019. Before July 2019, the atmosphere for waste sorting in the whole society was not strong, so social pressure and relatives and friends have little effect impact on residents. In July 2019, Xi Jinping, the president of China, raised waste sorting to a national strategy and subsequently, 46 cities throughout the country started pilot projects. Shanghai individual residents were gradually affected by social pressure and the influence of relatives and friends, which gradually had an important influence on their waste sorting intentions.

Perceived policy effectiveness significantly affects household attitudes and their intentions of waste sorting. It is consistent with the research conclusions of Shen et al. [1] Wan et al. [14] and Liao et al. [16] in terms of the relationship of intentions. However, the former two studies [1,14] argue that the understanding of policy effectiveness of residents is not significantly related to attitude. China is a typical “family country” society, where the government has good credibility, the public pays great attention to policies, has strong confidence in the effectiveness of the implementation of national policies, and the participation of society, especially the community, keeps increasing. In addition to the attention paid by national leaders to waste sorting then public attention arising, Shanghai entered the mandatory era of waste sorting in July 2019. There were frequent online episodes with all kinds of Internet jokes proliferating through social media such as WeChat and Tik-Tok, forming a situation of “Onlookers across the country watched Shanghai’s demonstration”, which to some extent also led to the residents’ perception of waste sorting related policies in China. Furthermore, the Shanghai government carried out a policy of mandatory garbage classification. All wastes are required to be divided into four bins with different colours and logos, respectively, “dry waste bin”, “wet waste bin”, “recyclable waste bin” and “hazardous waste bin”. Individuals or organisations that fail to dispose of waste correctly will be fined between 50 yuan and 50,000 yuan [3].

Individual characteristics have a significant positive impact on the decision making about waste sorting behaviour, verifying the conclusions of Shen et al. [1] and Xu et al. [17]. This is contrary to the study results of Ofstad et al. [12] that the casual paths in the model are no different across the subgroups. From the perspective of gender, there is a difference in the significance level of residents’ attitudes (ATT) to waste sorting intention (WSI), indicating that women’s attitudes towards waste sorting are more supportive. From the perspective of income, there are differences in the significance of resident attitudes (ATT) and perceived behaviour control (PBC) to waste sorting intention (WSI). Residents with higher income (RMB > 5000 per month) are more willing to adopt waste sorting. The intention to sort the waste of low-income (RMB < 5000 per month) residents is more likely to be affected by perceived behaviour control. From the perspective of age, the residents’ attitudes (ATT), the subjective norms (SN), the perceived behaviour control (PBC), and the perceived policy effectiveness (PPE) have different significance levels for waste sorting intentions (WSI), indicating that the elderly residents are more willing to sort waste. Young adults are more susceptible to the influence of families and friends, which may be related to the larger social circle of this age group. Additionally, people in this age group are more susceptible to self-behaviour control and perception of policies. From the perspective of educational background, except for the difference in the significance level of the residents’ perceived behaviour control (PBC) to the waste sorting intention (WSI), the other differences are not significant, indicating that the perception of the difficulty of waste sorting by residents without higher education is more likely to affect waste sorting intention. Combined with the horizontal comparison of the waste sorting intention (WSI) with the waste sorting behaviour (WSB), regardless of gender, age, income level, educational background, and living area, there is no significant difference between the intention to sort household waste and actual waste sorting behaviour. Unified and coordinated, the urban and rural residents of Jiaxing City generally tend to implement waste sorting, and the intention of sorting behaviour is clear.

In summary, women, high-income, elderly people are more willing to sort waste, and residents with no higher education are more likely to perceive the difficulty of waste sorting and have more obvious intentions for waste sorting behaviour. Among the variables, living area has little impact on the decision making about waste sorting behaviour, which may be related to the relatively developed economy of the sample location in Jiaxing City and the higher degree of urban-rural integration.

## 6. Conclusions, Limitations, and Future Research

This study uses an integrated model of the theory of planned behaviour (TPB) and value-belief-norm theory (VBN), taking Jiaxing City, Zhejiang Province of China as an example, to investigate the behavioural intention of household waste sorting and the factors affecting the waste sorting behaviour mechanism. After empirical analysis, the following conclusions are drawn: (1) Attitudes, subjective norms, perceived behaviour control, and perceived policy effectiveness have a significant positive impact on household waste sorting intentions, and household waste sorting intentions significantly determine their waste sorting behaviours; (2) subjective norms and perceived policy effectiveness are the main factors influencing household waste sorting intentions; (3) individual characteristics such as gender, age, and income have a significant impact on household waste sorting behaviour.

To promote the behaviour of household waste sorting of residents and promote further development of waste classification in China, this study put forward the following recommendations.

Firstly, we must effectively implement policies to increase residents’ awareness of the government’s waste sorting policy. In the process of policy implementation, it is necessary to achieve policy transparency and information disclosure, which can improve residents’ perception of the effectiveness of the policy, thereby increasing residents’ enthusiasm for waste sorting.

Secondly, a new fashionable social atmosphere for waste sorting needs to be formed and elderly educated women are encouraged to be evolved. It is necessary to pay full attention to the positive influence of attitudes of resident behaviour and subjective norms on waste sorting intention, increase the publicity of waste sorting, especially to play the role of “community aunts” in the community and educated elderly women, who are enthusiastic and knowledgeable, and more conducive to large-scale waste sorting campaigns. This culture of grassroots governance has a track record of success in China. Thus, a good publicity atmosphere for waste sorting in communities and various workplaces can be created with high-efficiency front-end sorting. This source sorting of garbage is the starting point for the entire waste sorting process.

Thirdly, it is fundamental to improve the construction of waste sorting facilities to enhance the convenience of household waste sorting. It is necessary to scientifically plan the layout of front-end classified release facilities, the operation of middle-end classified vehicles, and end-dry and wet-waste sorting and disposal facilities, etc., to enhance the sense of mission and responsibility of residents’ participation. Furthermore, Black technology helps waste sorting. Huawei has introduced AI apps for waste category inquiry. Some smart waste sorting and recycling systems such as intelligent bins have been applied in some cities. They have many functions, such as scanning QR codes for opening, closing, weighing, and settlement, etc. Accurate input can automatically score points, which can be used to exchange daily necessities for the residents.

Finally, waste sorting requires social co-governance. It is necessary to improve a complete legal system for waste sorting, form a government, NGO, and public–society co-governance system, make fine-grained regulations on the disposal, recycling, and disposal of waste sorting, and strengthen the operability and pertinence of laws and regulations. In short, waste sorting is a revolution in resident psychology and behaviour. Not only the government, but the entire society must take active actions to promote the development of a new fashion in waste sorting.

The first limitation of the study is the research design. This cross-sectional study has a likelihood of common source bias due to self-reported data. In the future, we can use eye-tracking measurement or brain-imaging tools to decrease self-reporting bias in creative behaviour research. The second research limitation is the sample of this study. The sample size of our study is relatively small (N = 541). Moreover, our results likely represent only the influencing factors of waste sorting behaviour intention in Jiaxing and likely cannot be generalized. Specifically, the degree of waste classification implementation in Jiaxing is low, which could hinder the generalizability of our study results. For applying the model to different environments and different groups, further research is necessary. Despite these limitations, our findings regarding the factors shaping residents’ intention and behaviours to classify waste should aid governments in their attempt to make breakthroughs in policy design and implementation.

Future research can empirically test the conceptual model of this study for investigating other green practices to improve sustainable development in China as well as other countries. From a psychological perspective, considering other individual characteristics in terms of intention behaviour, that are not included in this research, may also have the potential to extend the literature on waste management and/or other environmentally friendly practices.

## Figures and Tables

**Table 1:** Summary of the dimension measurement method.

Variables	Indicators	Contents	Sources
Attitude	ATT1	The waste sorting is a hygienic and healthy behaviour.	(Shen et al. [1]; Ajzen et al. [9]; Jia et al. [10])
	ATT2	The recycling of waste makes me feel responsible for environmental protection.	
	ATT3	Implementing waste sorting is a very good measure.	
	ATT4	For me, it’s very good to implement waste sorting regularly.	
	ATT5	Implementing Waste sorting regularly makes me happy.	
Subjective norms	SN1	My families think waste sorting is effective.	(Taylor and Todd [17]; Baber [18])
	SN2	People who are important to me support me with waste sorting.	
	SN3	I will do the same when I see people waste sorting around me.	
	SN4	My important friends/family think that waste sorting is very important for environmental protection.	
	SN5	My important friends/family recommended that I waste sort.	
Perceived behaviour control	PBC1	I have complete control over deciding whether to sort waste.	(Ajzen and Thomas [9]; Kim [11]; Ofstad et al. [12])
	PBC2	I can avoid polluting the living environment through waste sorting.	
	PBC3	I am willing to sort waste to protect the environment in the community.	
Perceived policy effectiveness	PPE1	The waste sorting and recycling bins provided by the government can promote recycling.	(Wan [15]; Liao et al. [16]; Gkargkavouzi et al. [20])
	PPE2	The environmental protection plan implemented by the government has effectively raised public awareness of environmental hazards.	
	PPE3	The government provides clear guidelines on waste sorting.	
	PPE4	The government’s propaganda helps citizens understand the importance of waste sorting.	
	PPE5	Government policy encourages me to sort the waste.	
	PPE6	The government policy is conducive to our waste sorting.	
	PPE7	The release of the policy makes me want to implement waste sorting.	
Waste sorting intention	IWS1	In the next few weeks, I plan to reduce food waste by paying more attention to the amount purchased.	(Ajzen and Thomas [9]; Ajzen [8]; Chu and Chiu [13])
	IWS2	After that, I plan to sort waste several times a week.	
	IWS3	From this week on I will sort the waste.	
	IWS4	I want to let my family and friends sort the trash.	
	IWS5	I am willing to learn waste sorting knowledge to better classify.	
Waste sorting behaviour	WSB1	I implemented the recycling sorting.	(Ofstad et al. [12])
	WSB2	I implemented hazardous waste sorting.	
	WSB3	I implemented the sorting of food waste.	
	WSB4	I implemented the sorting of other waste.	

**Table 2:** Factor loadings and cross loadings of the external model.

	ATT	SN	PBC	PPE	WSI	WSB
ATT1	0.758	0.618	0.585	0.546	0.556	0.298
ATT2	0.891	0.828	0.754	0.799	0.810	0.501
ATT3	0.863	0.742	0.723	0.764	0.749	0.462
ATT4	0.875	0.757	0.729	0.727	0.794	0.450
ATT5	0.805	0.753	0.612	0.612	0.685	0.402
SN1	0.604	0.741	0.474	0.549	0.560	0.351
SN2	0.755	0.822	0.589	0.641	0.667	0.385
SN3	0.812	0.805	0.725	0.736	0.759	0.463
SN4	0.791	0.881	0.694	0.774	0.784	0.495
SN5	0.580	0.776	0.528	0.641	0.705	0.485
PBC1	0.385	0.423	0.599	0.419	0.447	0.349
PBC2	0.725	0.660	0.889	0.685	0.696	0.402
PBC3	0.807	0.720	0.914	0.713	0.757	0.446
PPE1	0.615	0.647	0.643	0.791	0.666	0.424
PPE2	0.801	0.777	0.741	0.860	0.768	0.504
PPE3	0.637	0.658	0.597	0.837	0.706	0.507
PPE4	0.708	0.709	0.640	0.887	0.754	0.509
PPE5	0.679	0.661	0.572	0.720	0.619	0.351
PPE6	0.697	0.688	0.631	0.885	0.736	0.507
PPE7	0.722	0.757	0.640	0.878	0.782	0.530
WSI1	0.687	0.696	0.604	0.721	0.820	0.525
WSI2	0.623	0.670	0.643	0.670	0.831	0.524
WSI3	0.719	0.755	0.664	0.740	0.859	0.633
WSI4	0.799	0.786	0.742	0.774	0.895	0.534
WSI5	0.825	0.770	0.714	0.728	0.825	0.440
WSB1	0.493	0.501	0.461	0.548	0.608	0.909
WSB2	0.389	0.418	0.387	0.422	0.464	0.821
WSB3	0.453	0.514	0.425	0.530	0.588	0.881
WSB4	0.402	0.425	0.398	0.433	0.471	0.808

**Table 3:** The results of hypotheses testing.

Hypotheses Path	Path Coefficient (β)	T	Results
H1: ATT → WSI	0.198 ***	3.203	Supported
H2: SN → WSI	0.314 ***	5.926	Supported
H3: PBC → WSI	0.162 ***	4.115	Supported
H4: WSI → WSB	0.629 ***	18.513	Supported
H5: PPE → ATT	0.831 ***	45.384	Supported
H6: PPE → WSI	0.308 ***	6.396	Supported

Note: *** *p* < 0.001.

**Table 4:** The results of multi-group analysis (1).

	Gender		Income		Age	
	Male	Female	Low	High	Young	Elderly
	*n* = 274	*n* = 267	*n* = 365	*n* = 185	*n* = 414	*n* = 127
H1	0.180	0.214 **	0.155 *	0.349 ***	0.154 *	0.469 ***
H2	0.337 ***	0.296 ***	0.349 ***	0.200 ***	0.336 ***	0.220 *
H3	0.176 **	0.161 **	0.161 ***	0.146 *	0.177 ***	0.099
H4	0.575 ***	0.692 ***	0.617 ***	0.616 ***	0.614 ***	0.639 ***
H5	0.834 ***	0.828 ***	0.809 ***	0.874 ***	0.815 ***	0.884 ***
H6	0.281 ***	0.324 ***	0.310 ***	0.304 ***	0.315 ***	0.193 *

Note: * *p* < 0.05; ** *p* < 0.01; *** *p* < 0.001.

**Table 5:** The results of multi-group analysis (2).

	Education Background		Living Area	
	Non-Higher Education	Higher Education	Urban	Rural
	*n* = 319	*n* = 222	*n* = 365	*n* = 176
H1	0.202 **	0.221 *	0.180 *	0.219 *
H2	0.312 ***	0.322 **	0.318 ***	0.340 ***
H3	0.182 ***	0.114	0.163 **	0.158 **
H4	0.573 ***	0.654 ***	0.598 ***	0.693 ***
H5	0.796 ***	0.878 ***	0.826 ***	0.842 ***
H6	0.290 ***	0.316 ***	0.321 ***	0.270 ***

Note: * *p* < 0.05; ** *p* < 0.01; *** *p* < 0.001.

## Data Availability

The data used and analysed in this study are available on request from the corresponding author.

## References

[1] Shen X., Chen B., Leibrecht M., Du H. (2022). The Moderating Effect of Perceived Policy Effectiveness in Residents’ Waste Classification Intentions: A Study of Bengbu, China. Sustainability.

[2] Lu X., Pu X., Han X. (2022). Sustainable smart waste classification and collection system: A bi-objective modeling and optimization approach. J. Clean. Product..

[3] Tong Y., Liu J., Liu S. (2019). China is implementing “garbage classification” action. Environ. Pollut..

[4] Zhang Y., Wang G., Zhang Q., Ji Y., Xu H. (2022). What determines urban household intention and behavior of solid waste separation? A case study in China. Environ. Imp. Assess. Rev..

[5] Yang J., Long R., Chen H., Sun Q. (2021). A comparative analysis of express packaging waste recycling models based on the differential game theory. Resour. Conserv. Recycl..

[6] Pekarkova Z., Williams I., Emery L., Bone R. (2021). Economic and climate impacts from the incorrect disposal of WEEE. Resour. Conserv. Recycl..

[7] Alhassan H., Kwakwa P.A., Owusu-Sekyere E. (2019). Households’ source separation behaviour and solid waste disposal options in Ghana’s Millennium City. J. Environ. Manag..

[8] Ajzen I. (1991). The Theory of Planned behaviour. Organ. Behav. Hum. Decis. Processes.

[9] Ajzen I., Madden T.J. (1986). Prediction of goal-directed behaviour: Attitudes, intentions, and perceived behavioural control. J. Exper. Soc. Psychol..

[10] Jia Y., Cheng S., Shi R. (2021). Decision-making behavior of rural residents’ domestic waste classification in Northwestern of China: Analysis based on environmental responsibility and pollution perception. J. Clean. Prod..

[11] Kim Y. (2010). An investigation of green hotel customers’ decision formation: Developing an extended model of the theory of planned behaviour. Int. J. Hosp. Manag..

[12] Ofstad S.P., Tobolova M., Nayum A., Klöckner C.A. (2017). Understanding the Mechanisms behind Changing People’s Recycling Behavior at Work by Applying a Comprehensive Action Determination Model. Sustainability.

[13] Chu P.Y., Chiu J. (2003). Factors Influencing Household Waste Recycling behaviour: Test of an integrated Model. J. Appl. Soc. Psychol..

[14] Wan C., Shen G., Yu A. (2014). The role of perceived effectiveness of policy measures in predicting recycling behaviour in Hong Kong. Resour. Conserv. Recycl..

[15] Granco G., Heier Stamm J., Bergtold J., Daniels M.D., Sanderson M.R., Sheshukov A.Y., Mather M.E., Caldas M.M., Ramsey S.M., Lehrter R.J. (2019). Evaluating environmental change and behavioural decision-making for sustainability policy using an agent-based model: A case study for the Smoky Hill River Watershed, Kansas. Sci. Total Environ..

[16] Liao C., Zhao D., Zhang S., Chen L. (2018). Determinants and the Moderating Effect of Perceived Policy Effectiveness on Residents’ Separation Intention for Rural Household Solid Waste. Int. J. Environ. Res. Public Health.

[17] Xu L., Ling M., Shen M. (2017). External influences on forming residents’ waste separation behaviour: Evidence from households in Hangzhou, China. Habitat Int..

[18] Taylor S., Todd P.A. (1995). Understanding Information Technology Usage: A Test of Competing Models. Inform. Sys. Res..

[19] Baber H. (2019). Subjective Norms and Intention-A Study of Crowdfunding in India. Res. World Econ..

[20] Gkargkavouzi A., Halkos G., Matsiori S. (2019). Environmental behaviour in a private-sphere context: Integrating theories of planned behaviour and value belief norm, self-identity, and habit. Resour. Conserv. Recycl.

[21] Park J., Ha S. (2014). Understanding Consumer Recycling behaviour: Combining the Theory of Planned behaviour and the Norm Activation Model. Fam. Consum. Sci. Res. J..

[22] Nuyen T., Zhu D., Le N. (2015). Factors influencing waste separation intention of residential households in a developing country: Evidence from Hanoi, Vietnam. Habitat Int..

[23] Fornell C., Larcker D.F. (1981). Evaluating structural equation models with unobservable variables and measurement error. J. Mark. Res..

[24] Hair J.F., Sarstedt M., Ringle C.M., Mena J.A. (2012). An Assessment of the Use of Partial Least Squares Structural Equation Modeling in Marketing Research. J. Acad. Mark. Sci..

[25] Hair J., Sarstedt M., Pieper T., Ringle C. (2012). The Use of Partial Least Squares Structural Equation Modeling in Strategic Management Research: A Review of Past Practices and Recommendations for Future Applications. Long Rang. Plan..

[26] Khan G., Sarstedt M., Shiau W., Hair J., Ringle C., Fritze M. (2019). Methodological research on partial least squares structural equation modeling (PLS-SEM). Internet Res..

[27] Ringle C.M., Wende S., Becker J.M. (2015). SmartPLS 3.3.3.

[28] Hair J.F., Risher J.J., Sarstedt M., Ringle C.M. (2019). When to use and how to report the results of PLS-SEM. Eur. Bus. Rev..

